# Antibiotic consumption and antimicrobial resistance in Poland; findings and implications

**DOI:** 10.1186/s13756-018-0428-8

**Published:** 2018-11-15

**Authors:** Jadwiga Wojkowska-Mach, Brian Godman, Amanda Glassman, Amanj Kurdi, Andrzej Pilc, Anna Rozanska, Szymon Skoczyński, Marta Wałaszek, Tomasz Bochenek

**Affiliations:** 1 Department of Microbiology, Faculty of Medicine, Jagiellonian University Medical College, Czysta 18 Str., 31-121, Krakow, Poland; 2 Strathclyde Institute of Pharmacy and Biomedical Sciences, Strathclyde University, G4 ORE, Glasgow, UK; 3 Health Economics Centre, Liverpool University Management School, Chatham Street, Liverpool, UK; 4 Department of Laboratory Medicine, Division of Clinical Pharmacology, Karolinska Institute, Karolinska University Hospital Huddinge, SE-141 86 Stockholm, Sweden; 5 Department of Public Health Pharmacy and Management, School of Pharmacy, Sefako Makgatho Health Sciences University, Garankuwa, South Africa; 6 Center for Global Development, 2055 L Street NW, Washington, DC 20036 USA; 7 Department of Pharmacology, College of Pharmacy, Hawler Medical University, Erbil, Iraq; 8 Department of Neurobiology, Institute of Pharmacology, Polish Academy of Sciences, Krakow, Poland; 9 Department of Drug Management, Faculty of Health Sciences, Jagiellonian University Medical College, Grzegorzecka 20 Str., 31-531, Krakow, Poland; 10 Department of Pneumonology, School of Medicine in Katowice, Medical University of Silesia, Katowice, Poland; 11 Department of Nursing, Institute of Health Sciences, State Higher Vocational School in Tarnów, Tarnów, Poland

**Keywords:** Antibiotic consumption, Antimicrobial resistance, Antimicrobial resistance surveillance, European Union, Health policy, Poland

## Abstract

**Background:**

The problem of inappropriate use of antibiotics and the resulting growth in antimicrobial resistance (AMR) has implications for Poland and the world. The objective of this paper was to compare and contrast antibiotic resistance and antibiotic utilisation in Poland in recent years versus other European countries, including agreed quality indicators, alongside current AMR patterns and ongoing policies and initiatives in Poland to influence and improve antibiotic prescribing.

**Methods:**

A quantitative ten-year analysis (2007–2016) of the use of antibiotics based on European Centre for Disease Prevention and Control (ECDC) data combined with a literature review on AMR rates and antimicrobial stewardship initiatives.

**Results:**

The system of monitoring AMR and appropriate strategies to address AMR rates remain underdeveloped in Poland. The role of microbiological diagnostics and efforts to prevent infections is currently underestimated by physicians. Overall, Poland had one of the highest rates of total consumption of antibiotics in the analysed European countries. Total consumption of antibacterials for systemic use and relative consumption of beta-lactamase sensitive penicillins were characterized by small but statistically significant average annual increases between 2007 and 2016 (from 22.2 DIDs to 23.9 DIDs and from 0.8 to 1.3%, respectively).

**Conclusions:**

The integrated activities around appropriate antibiotic prescribing in the pre- and post-graduate training of physicians and dentists seem to be particularly important, as well as changes in policies on prescribing antibiotics within ambulatory care. AMR and appropriate prescribing of antibiotics should be the focus of health policy actions in Poland.

## Background

### General

Antibiotics have significantly improved the prognoses of patients with infectious diseases, reducing morbidity and mortality. However, the use of antibiotics is invariably associated with the risk of resistance development, with numerous studies confirming the association between increasing use of antibiotics and enhanced antimicrobial resistance (AMR) [1–6]. Increasing AMR rates are not helped by concerns regarding the choice of antibiotic or duration of therapy, which can be incorrect in up to 50% of cases [3, 7], as well as the extent of irrational prescribing of antibiotics for essentially viral infections [3], or the increasing use of antibiotics in agriculture [7]. Acute upper respiratory tract infections (URTIs), which are the most common infections in ambulatory care, are essentially viral in origin but with high rates of inappropriate antibiotic use across countries [3, 8–10].

As a result of growing concerns, we are seeing an increasing number of programmes and activities across countries, including European countries, to enhance appropriate antibiotic use to reduce AMR rates. These include the development of Antimicrobial Stewardship Program (ASP), as well as educational activities among physicians and pharmacists [8, 11–17]. ASPs include analysis of the consumption of antibiotics over time alongside instigation and monitoring of activities to improve appropriate use.

However, whilst the European Surveillance of Antimicrobial Consumption (ESAC) network, and more recently the European Centre for Disease Prevention and Control (ECDC) [18, 19] and WHO Europe [20], have researched antibiotic utilisation across Europe including Poland, as well as assessed quality indicators for antibiotic utilisation, there is currently limited patient-level data to improve antibiotic prescribing in Poland. Physicians are one of the key stakeholders to improve future antibiotic use in Poland, with limited if any purchasing of antibiotics without a prescription due to current restrictions.

We are aware of concerns with antibiotic prescribing in Poland. A study published in 2008 showed that between 2002 and 2005, at least 64.3% of the studied population were prescribed antibiotics [21]. Panasiuk et al. reported that 78.5% of adults with acute URTIs had antibiotics as empirical first-line treatment [22]. Overall anti-infectives (Anatomical Therapeutic Chemical class: ATC J01-J07X) constituted approximately 5% of pharmaceutical reimbursement in ambulatory care in Poland in 2010, with amoxicillin with clavulanic acid among the top 25 reimbursed medicines with the greatest sales [23]. However, between 2004 and 2008, Poland’s total antibiotic use was comparable to the median European level [24].

There have been initiatives in Poland to improve physician antibiotic prescribing through guidelines, with the most recent guidelines published in 2016 [25]. However, there have been concerns whether these have been properly disseminated and implemented, especially given current individualism within the Polish healthcare system, acceptance of authority (‘power distance’), and physicians wishing to avoid uncertainty [26]. In addition, current short medical consultations do not allow for establishing a close relationship between physicians and their patients, including educating patients regarding the appropriate use of antibiotics. There are also concerns with the lack of National Health Fund (NHF) activity to improve the prescribing of antibiotics among physicians in Poland unlike co-ordinated activities in, for instance, the Republic of Srpska [14], as well as former Soviet Union Republics, such as Azerbaijan [17].

There are also concerns with the rate of hospital acquired infections in Poland [27], which need addressing alongside generally improving antibiotic utilization in Poland to reduce AMR rates.

### Antimicrobial resistance patterns across Europe and activities to reduce AMR

The gathering and analysis of information on drug resistance of bacterial strains among European countries is facilitated by the Healthcare-Associated Infections Surveillance Network (HAI-Net) [28] within the European Centre for Disease Prevention and Control (ECDC). Detailed data on drug resistance of selected bacterial strains and groups of antibiotics are gathered within the European Antimicrobial Resistance Surveillance Network (EARS-Net) [29].

As discussed, there are several ongoing activities across countries to reduce rising AMR rates given ongoing concerns. Identified objectives within the WHO Global Plan on Antimicrobial Resistance [30] included sharing knowledge and evidence, as well as instigating programmes to optimise antibiotic use through education and other activities. This is particularly important in Poland, where the incidence of various types of infections, including surgical site infections [31] and bloodstream infections associated with central venous catheters, appear higher than in neighbouring countries [27].

We are aware there needs to be greater documentation and discussions about current activities in Poland to improve antibiotic use. This includes assessing the quality of antibiotic use in ambulatory care against agreed indicators, including the use of combination penicillins, third- and fourth-generation cephalosporins, as well as fluoroquinolones, as these are considered by the WHO and others as critically important antibiotics to monitor [20, 32, 33].

Consequently, the objective of this paper was to compare and contrast antibiotic resistance and antibiotic utilisation in Poland in recent years versus other European countries, including agreed quality indicators, alongside current AMR patterns and ongoing policies and initiatives in Poland to influence antibiotic prescribing. The findings will help guide the development of future pertinent policies to improve antibiotic prescribing in Poland, given current concerns.

## Methods

A quantitative research was conducted on ECDC findings and combined with a literature review to document current antibiotic resistance rates in Poland, as well as initiatives to improve antibiotic prescribing. The literature review was not a systematic review; however, it included most cited expert publications. Simple statistical analyses were performed to help identify any trends where pertinent. We have used such approaches in previous publications to assess the influence of current antibiotic strategies as well as help plan future activities [13, 14, 16, 17]. Table 1 presents the measures which were used to assess the quality of antibiotic prescribing in Poland, in ambulatory care, during the past 10 years (i.e. from 2007 to 2016) based on ECDC, WHO and other suggested indicators among the J01 ATC class [19, 21, 32–34]. Linear regression analysis was used to assess any significant changes in the trends of the quality indicator measures (continuous variable) over the study period from 2007 to 2016; hence five regression models were conducted; one model per each quality indicator measure, but with time as the independent factor in all of the five models.

**Table 1:** Indicators used to assess the quality of antibiotic prescribing

Indicator’s description	Indicator’s acronym or shortened name
Consumption of antibacterials for systemic use (J01) expressed in defined daily doses per 1000 inhabitants per day (DIDs)	Total consumption J01
Consumption of beta-lactamase sensitive penicillins (J01 CE) expressed as percentage of the total consumption of antibacterials for systemic use (J01); i.e. relative consumption of beta-lactamase sensitive penicillins	J01 CE%
Consumption of combination of penicillins, including beta-lactamase inhibitor (J01CR) expressed as percentage of the total consumption of antibacterials for systemic use (J01); i.e. relative consumption of combination of penicillins, including beta-lactamase inhibitor	J01CR%
Consumption of third- and fourth-generation cephalosporins (J01(DD + DE)) expressed as percentage of the total consumption of antibacterials for systemic use (J01); i.e. relative consumption of third- and fourth-generation cephalosporins	J01DD + DE%
Consumption of fluoroquinolones (J01MA) expressed as percentage of the total consumption of antibacterials for systemic use (J01); i.e. relative consumption of fluoroquinolones	J01MA%
Ratio of the consumption of broad-spectrum (J01(CR + DC + DD+(F-FA01))) to the consumption of narrow-spectrum penicillins, cephalosporins and macrolides (J01(CE + DB + FA01))	J01B/N%

Ambulatory care, including general practitioner prescribing and out-patient dispensing (children and adult patients), was chosen for comparative purposes, as this contains the highest proportion of antibiotic use in Poland. In 2016, antibiotic consumption was 23.98 Defined Daily Doses per 1000 inhabitants per day (DIDs) within ambulatory (community) care, while only 1.36 DIDs within hospital sector [19]. In addition, comparative figures are available across Europe in recent years via ECDC. The only exceptions are Cyprus and Romania, where the authorities provided total consumption data, i.e. including in-patient use, as they were unable to separate out the different components.

The findings for Poland in 2016 were also compared with other European countries including those with the highest and lowest values for each indicator, with a special emphasis on neighbouring CEE countries, to again provide future guidance. Descriptive statistics were used to summarise the study variables. Univariate linear regression was used to evaluate any annual trend change in the study outcomes during the study period.

## Results

### Specific actions to prevent AMR in Poland

The coordinated system of monitoring AMR rates among patients in Poland has been implemented since 1997 with the establishment of the National Reference Centre for Antimicrobial Susceptibility Testing (NRCAST). Since 2002, information on antimicrobial utilisation across sectors, including hospitals and ambulatory care, has also been gathered via NRCAST. This organisation is also in charge of identification and susceptibility testing of bacterial strains from serious and difficult to diagnose infections, with a special focus on certain pathogens such as Methicillin-resistant *Staphylococcus aureus* (MRSA), and monitoring of the spread of resistant strains in hospitals and ambulatory care across Poland. NRCAST also provides data to the European Antimicrobial Resistance Surveillance Network (EARSS-Net) as well as ECDC.

In 2004, the Ministry of Health (MoH) established the National Programme for the Protection of Antibiotics (NPPA) coordinated by the National Institute of Medicines. with the main goal: to monitor AMR in Poland. The MoH is also the main stakeholder in charge of policy development to help prevent or reduce AMR in Poland as well as providing the legal framework for hospital infection control. The Chief Sanitary Inspectorate is responsible for suggesting potential initiatives where concerns exist. In practice, activity of the MoH in the area of AMR monitoring and prevention has been limited, principally left to the State Consultant for Microbiology and the Chief Sanitary Inspectorate to formulate policies on AMR. As far as the NHF’s involvement is concerned, usually only limited activities have been undertaken to date to help improve antibiotic use with appreciable autonomy among physicians. Having said this, pneumococcal vaccination became compulsory in Poland in January 2017. The roles of other Polish health care system stakeholders in policy formulation to prevent or reduce AMR are principally advisory ones.

### Current resistance patterns in Poland

Infections in Polish patients illustrate the relationship between high utilisation and high levels of resistance, i.e. the high consumption of trimethoprim and sulfamethoxazole [35] has been accompanied by high levels of antibiotic resistance [5]. Having said this, the prevalence of MRSA in Poland in 2013 was 16%, with prevalence rates exceeding 25% in many EU Member States [35].

Concerns with current AMR rates in Poland are confirmed by data on *Acinetobacter baumannii* infection (ACI), which are some of the most important opportunistic pathogens responsible for the most severe infections characterised by the high level of resistance to commonly used antibiotics. More than 75% of ACI strains of *A. baumannii* isolated from pneumonia cases among hospitalised patients in southern Poland were resistant to 14 out of 16 antimicrobials tested [36], with approximately 60% of *A. baumannii* strains from patients with invasive infections, pneumonia, bloodstream infections and meningitis, resistant to all antimicrobials tested with the exception of colistin. The extensively-drug resistant strains accounted for 80.8% of isolates tested [37]. The largest AMR outbreak in Poland in recent years was observed between 2012 and 2014 in two major cities (Warsaw and Poznan). It concerned New Delhi metallo-β-lactamase (NDM)-producing Enterobacteriaceae. Out of 374 cases of infection/colonisation with NDM-positive Enterobacteriaceae identified in that period, 370 cases were associated with a *Klebsiella pneumoniae* outbreak [38].

Concerns with AMR are exacerbated by the fact that the current surveillance systems in Poland do not currently function optimally. Examples include concerns with data on MRSA epidemiology both in the general population of hospitalised patients, as well as new-borns with very low birth weight [39]. Interestingly, along with a high level of MRSA prevalence at 15% among adult patients in general hospitals, considerable heterogeneity of strains has been detected.

### Empiric use of antibiotics vs. routine testing as well as prevention

Efforts in Poland to contain AMR should ideally rely on microbiological tests to guide current treatment as well as provide guidance on optimal empiric treatment when the need arises. A good example is the Polish Neonatological Network where data is gathered from Neonatal Intensive Care Units (NICU) on infection surveillance among newborns with very low birth weights [39]. The analysis of antibiotic utilisation in MRSA infections compared with methicillin-sensitive *Staphylococcus aureus* (MSSA) infections revealed that average therapy durations were 11.2 and 12.3 days respectively [39]. Concurrently, the level of glycopeptides’ use was high in both MRSA and MSSA infections, providing a rationale for the introduction of quick molecular tests into routine clinical practice, especially in NICUs [40].

Despite situations such as this, the role of microbiological diagnostics to reduce empiric use of antibiotics as well as efforts to prevent infection is currently underestimated by physicians in Poland [38, 41, 42]. Nowadays, there is a greater focus on treatment rather than prevention, since in Poland, unlike some Western European countries, diagnostic laboratories are organised separately from hospital structures, with most microbiologists not directly involved in treatment decisions. This is particularly problematic for nosocomial infections.

In addition, usually health care providers (hospitals, family physicians) must pay for microbiologic diagnostic tests from their budgets and ambulatory care patients must pay for tests out of pocket. Both of these can be a concern. Consequently, most community acquired infections are currently treated empirically.

### Quantitative analysis of antibiotic prescribing quality indicators in ambulatory care

Table 2 contains details of the pertinent antibiotic prescribing quality indicators in ambulatory care in Poland during the past 10 years. Total consumption of antibacterials for systemic use and consumption of fluoroquinolones expressed as percentage of the total consumption of antibacterials for systemic use (i.e. relative consumption of fluoroquinolones) were characterized by small increases between 2007 and 2016 (8.3 and 13.5%, respectively), with a significant average annual increase of 3.6% (*p* = 0.0357) for total antibiotic consumption. Relative consumption of beta-lactamase sensitive penicillins revealed a much bigger increase in the analyzed period (62.5%), with a significant average annual increase of 0.0006% (*p* = 0.0391). The relative consumption of combination of penicillins, including beta-lactamase inhibitor, as well as a ratio of the consumption of broad-spectrum to the consumption of narrow-spectrum penicillins, cephalosporins and macrolides decreased in the analyzed period by 1.07 and 14.9% respectively.

**Table 2:** Key ambulatory care antibiotic prescribing quality indicators for Poland 2007 to 2016

Indicator – ECDC code	2007	2008	2009	2010	2011	2012	2013	2014	2015	2016	*P*-value*	% change 2016/2007
Total utilisation J01	22.2	20.8	23.6	21.1	21.1	22.9	23.6	22.8	26.2	23.9	0.0357	8.3
J01 CE%	0.8%	0.8%	0.6%	0.7%	0.5%	0.7%	0.7%	0.9%	1.3%	1.3%	0.0391	62.5
J01CR%	18.7%	21.7%	20.9%	21.3%	30.3%	16.9%	16.5%	16.6%	17.0%	18.5%	0.2949	−1.07
J01DD + DE%	< 0.1%	< 0.1%	< 0.1%	< 0.1%	< 0.1%	< 0.1%	< 0.1%	< 0.1%	< 0.1%	< 0.1%		NA
J01MA%	5.2%	5.8%	5.3%	5.8%	5.6%	5.3%	5.0%	5.3%	5.3%	5.9%	0.9867	13.5
J01B/N%	30.3%	32.5%	36.3%	37.1%	57.6%	36.9%	34.9%	29.0%	24. 8%	25.8%	0.3771	−14.9

Note: *p-value obtained from the linear regression model

Legend: explanation of acronyms is provided in Table 1

**Fig. 1:**
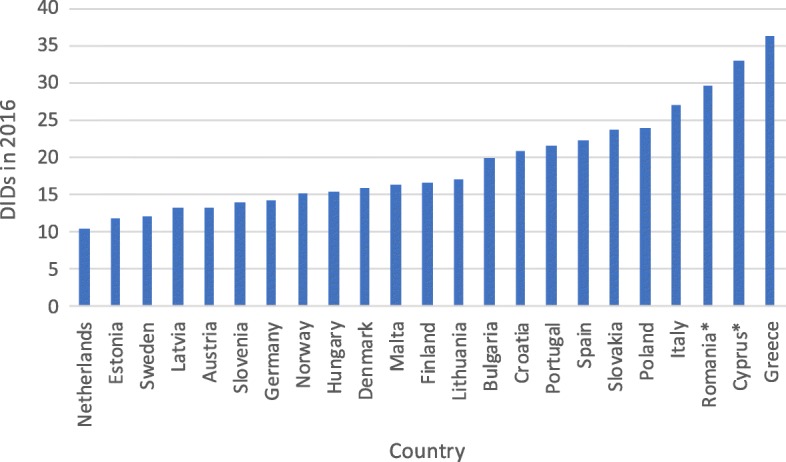
Ambulatory care antibiotic consumption across Europe in 2016 in DIDs. Legend: DIDs - Defined Daily Doses per 1000 inhabitants per day; *only total consumption data available

The total consumption of antibiotics in the surveyed European countries ranged in 2016 from 10 DIDs in the Netherlands to 36 DIDs in Greece (median 12 DIDs), while Poland had one of the highest consumption rates, exceeding the European median (expressed in DIDs) more than twice (Fig. 1). Only some countries of Southern Europe had higher consumption rates, while in the remaining 78.3% countries the consumption rates were lower than in Poland.

**Fig. 2:**
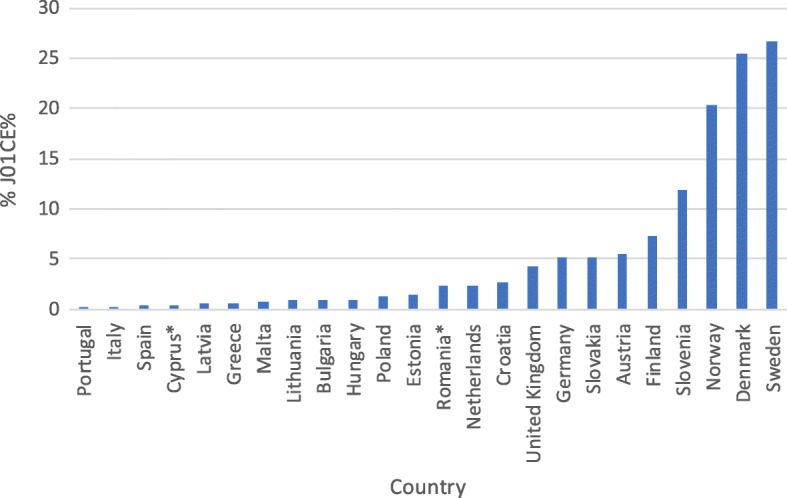
Consumption of beta-lactamase sensitive penicillins (J01 CE) expressed as percentage of total antibacterial consumption in 2016

**Fig. 3:**
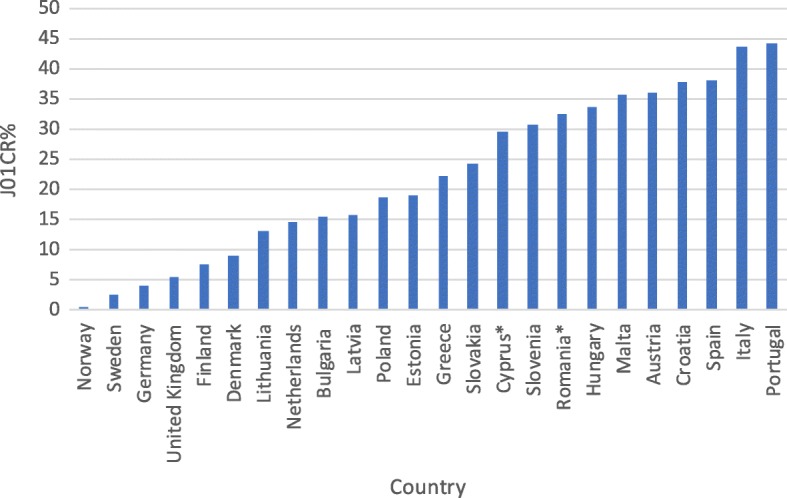
Consumption of combination penicillins, including beta-lactamase inhibitors (J01CR), expressed as percentage of total antibacterial consumption in 2016

**Fig. 4:**
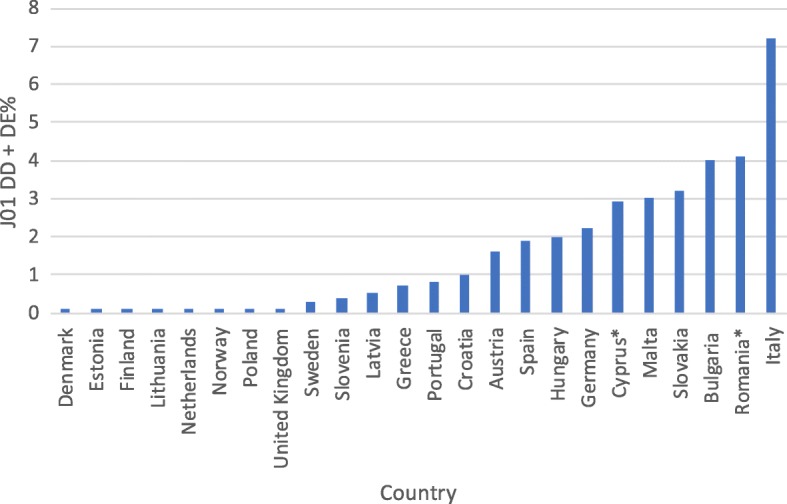
Consumption of third- and fourth-generation cephalosporins (J01DD + DE) expressed as percentage of total antibacterial consumption in 2016

**Fig. 5:**
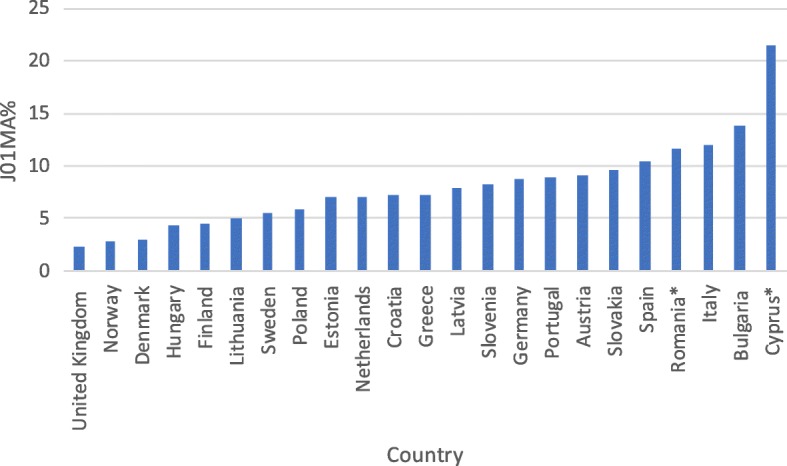
Consumption of fluoroquinolones (J01MA) expressed as percentage of total antibacterial consumption in 2016

**Fig. 6:**
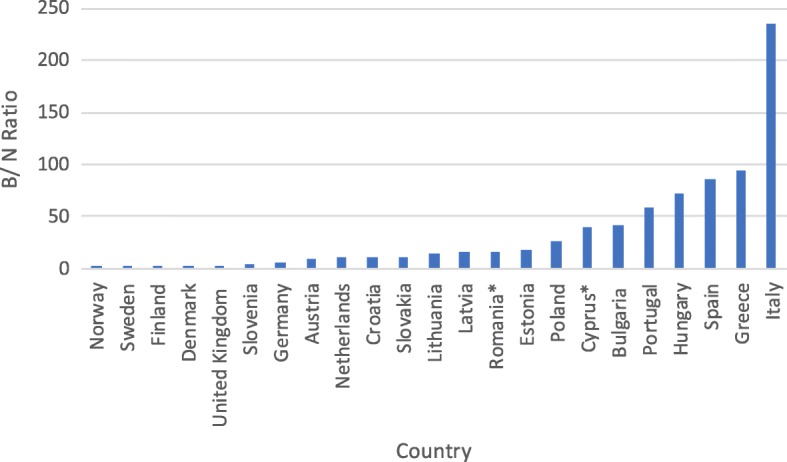
Consumption of broad-spectrum (B) to narrow-spectrum (N) antibiotics in 2016 (ratio B vs N)

However, interpretation of the other assessed indices does not bring unequivocally negative conclusions. There has been a sizeable increase in consumption of beta-lactamase sensitive penicillins (J01 CE) from a very low share of total antibiotic consumption in previous years (Table 2). The current share of these antibiotics in treatment is on a notably low level when compared with other European countries (Fig. 2). Encouragingly, the consumption of combination penicillins (J01CR), third- and fourth-generation cephalosporins (J01(DD + DE)) and fluoroquinolones (J01MA) is on a relatively low level (Figs. 3, 4, 5). Poland is among the European countries with the lowest share of these antibiotics within overall antibiotic consumption, especially with regard to third- and fourth-generation cephalosporins. On the other hand, in ambulatory care wide-spectrum antibiotics are used relatively more often (ratio J01(CR + DC + DD+(F-FA01) to J01(CE + DB + FA01)) – Fig. 6 (explanation of acronyms is provided in Table 1, see also Abbreviations). However, the situation has improved in comparison to the past years. In 2007 the share of wide-spectrum antibiotics was 30.3%, while in 2016 it was 25.8% (Table 2).

## Discussion

There is currently a variable situation regarding antibiotic prescribing in Poland. Poland is at the high end of antibiotic utilization in recent years; at almost 24 DIDs in 2016, appreciably higher than Estonia, Latvia or Slovenia. In addition, consumption grew significantly by about 8% between 2007 and 2016; however, appreciably lower than the 36% increase in total worldwide antibiotic consumption during the past decade [6]. There are also concerns with the low level of prescribing of beta-lactamase sensitive penicillins in Poland, compared for instance with Slovakia, Finland, Slovenia or Sweden. However, encouragingly prescribing rates for these penicillins have been growing in recent years. Encouragingly as well, there was relatively low consumption of combination penicillins and third- and fourth-generation cephalosporins in Poland, with a slight decrease over the years.

There was also a relatively low level of fluoroquinolone consumption in Poland versus other European countries (at 5.9% in 2016). However, there are concerns regarding the growth of fluoroquinolone prescribing in Poland in recent years, expressed as a percentage of the total antibiotic consumption (Table 2). The use of fluoroquinolones, due to their numerous side effects, should be limited to situations where there are no other therapeutic options available. One of the most important side effects of the use of fluoroquinolones is (among others) *Clostridium difficile* infections (CDI) [11]. The Polish hospitals reported almost the highest crude incidence density of community-associated CDI incidence densities (1.4 cases/10,000 patient-days) in Europe (EU/EEA mean was only 0.8 cases/10,000 patient-days) [43]. Consequently, there is an urgent need to educate physicians in Poland about the appropriate use of fluoroquinolones, unless this is a consequence of high antimicrobial resistance in Poland to other antibiotics. This will be investigated further in future research projects.

There are also concerns at the extent of prescribing of broad versus narrow spectrum antibiotics in Poland, their consumption ratios in 2016 being very high level at 25.81% of total antibiotic consumption versus for instance 0.68% in Finland. Fortunately, this ratio is decreasing in Poland.

This variable picture regarding current antibiotic utilisation patterns in Poland may well reflect the relative lack of educational activities by the NHF and the MoH among physicians. We do know that there is a lack of preparedness among physicians in Poland for educational activities in the field of rational antibiotic therapy. In addition, we are aware that research in area of medical education has shown that problem-based learning curricula are rarely introduced in a country with a high ‘power distance’ and ‘uncertainty avoidance’, which currently includes Poland [44]. This is confirmed by both high total consumption antibiotics versus other European countries and a high proportion (ratio) of broad-spectrum antibiotics. The notion of power distance refers to the degree of hierarchy in a country and it has been defined by Hofstede as the extent to which the less powerful members of organizations and institutions accept and expect that power is distributed unequally [45]. The notion of uncertainty avoidance deals with a society’s tolerance for uncertainty and ambiguity. A higher score indicates that people feel uncomfortable in novel, unknown or surprising situations [45].

Overall, integrated activities around appropriate antibiotic prescribing in the pre- and post-graduate training of physicians and dentists should be particularly important. Currently, the concept of antimicrobial stewardship (AMS) is not included in the curricula of studies and specializations except for specialization for microbiologists. However, in the Polish health care system there are very few physicians-microbiologists (only 110 professionally active in 2018). There could also be the involvement of other specialists in hospitals, such as hospital pharmacists, as part of Drugs and Therapeutic Committees helping with the implementation and monitoring of AMS programs [46].

Other direct measures, which could be recommended for implementation in Poland, include the introduction of limitations in antibiotic prescribing within ambulatory care in Poland, through their stratification, similar to the Republic of Srpska (Bosnia and Herzegovina) or Slovenia, or a basic positive list of medicines with reference prices and a supplementary list which includes more expensive medicines [13, 16]. In the Republic of Srpska amoxicillin, benzathine-phenoxymethylpenicillin, cefalexin, doxycycline, erythromycin, phenoxymethylpenicillin, and sulfamethoxazole with trimethoprim are reimbursed between 90 to 100%; with norfloxacin reimbursed 50%; with 100% co-payment for all other antibiotics [16]. In Slovenia, the national health insurance company has instigated prescribing restrictions for amoxicillin/clavulanic acid, third-generation cephalosporins, fluoroquinolones and the macrolides together with their place in therapy i.e., first, second or third line, to enhance antibiotic prescribing [13]. Such activities can potentially be applied in Poland to reduce excessive prescribing of broad-spectrum antibiotics.

Promoting and spreading knowledge on antibiotics, as medicines designated for combatting bacterial infections but ineffective against viruses, is urgently needed. The education campaigns should be targeted not only at physicians, but also patients, especially because other factors should be taken into consideration. These include the relative cost of prescribed antibiotic and the cost of medical consultations. Educational programmes, including academic detailing and providing decision support systems, have improved antibiotic use, although some of the results have been modest [8, 47, 48]. Feedback to physicians, especially high prescribers of antibiotics, has also worked well [49]. Enhancing physician communication skills, as well as the availability of point-of-care testing, can also reduce inappropriate prescribing of antibiotics for acute respiratory tract infections [50, 51]. Although not confirmed by prospective studies, it might be expected that in countries where medical consultations and the cost of antibiotics is high, there will be a lower likelihood for excessive use of antibiotics. Unfortunately, from this perspective, prices of commonly prescribed antibiotics are usually low in Poland, which may result in increased consumption and secondary antibiotic resistance. In contrast, ambulatory patients usually must pay out-of-pocket for microbiological diagnostic tests, which are very often more expensive than prescribed antibiotics. This may result in underutilization of diagnostic testing due to financial reasons.

Consequently, other avenues will be important for improving antibiotic utilization. The provision of health services is overwhelmingly focused on the treatment of infections rather than their prevention in Poland. This applies to many elements of infection control programmes, regarding both nosocomial and non-hospital infections. Notably, hand hygiene should be mentioned, since the existing level of knowledge of its rules is not sufficient among medical personnel and the compliance with recommendations is rather low in Poland. Intensive educational campaigns, addressed to both medical and social workers, are highly required. The more specific procedures, aiming to prevent certain forms of infections (e.g. ‘bundle strategies’ for prevention of device-associated infections in intensive care units, including small sets of evidence-based practices which have been proven to improve patient outcomes, when applied together) should be actively promoted and implemented. A more effective interdisciplinary education and cooperation between physicians and staff of microbiological laboratories are necessary, and implementation of fast and modern diagnostic methods is crucial. AMR surveillance is largely underdeveloped, and the scale of AMR in Poland may be underestimated. This is despite a relatively high consumption of antibiotics and some alarming trends for certain bacteriological strains. These are considerations for the future.

## Conclusions

Whilst the coordinated system of monitoring AMR rates in Poland was initiated almost 20 years ago, it remains underdeveloped, similarly to appropriate strategies to address AMR. The role of microbiological diagnostics, and efforts to prevent infections, is currently underestimated by physicians, who put a greater emphasis on treatment rather than prevention.

Total consumption of antibacterials for systemic use and relative consumption of beta-lactamase sensitive penicillins were characterized by small but statistically significant average annual increases between 2007 and 2016. Overall, Poland has one of the highest rates of total consumption of antibiotics among European countries. Although indicators such as the prevalence of MRSA are within the average values observed in the EU, in case of *P. aeruginosa*, *K. pneumoniae* and *Acinetobacter* there is over 50% resistance to fluoroquinolones, in invasive infections cases in Poland (highest prevalence rates among the EU countries).

Since the limited educational activities among physicians and dentists may play an important role in the current antibiotic consumption patterns, integrated actions focusing on appropriate antibiotic prescribing in the pre- and post-graduate training should be instigated and followed up at national level. The introduction of limitations in antibiotic prescribing within ambulatory care, through their stratification, is also recommended. Education campaigns targeted also to patients are urgently needed as well. Overall, taking actions to improve antibiotic utilisation should be in focus of health policy actions in Poland.

## Abbreviations

AMR: antimicrobial resistance
AMS: antimicrobial stewardship
ASP: Antimicrobial Stewardship Program
ATC: Anatomical Therapeutic Chemical
CDI: *Clostridium difficile* infections
CEE: Central and Eastern European
DDD: defined daily dose
DIDs: DDDs per 1000 inhabitants per day
EARSS-Net: European Antimicrobial Resistance Surveillance Network
ECDC: European Centre for Disease Prevention and Control
ESAC: European Surveillance of Antimicrobial Consumption
HAI-Net: Healthcare-Associated Infections Surveillance Network
J01 CE: beta-lactamase sensitive penicillins
J01(CE + DB + FA01): narrow-spectrum penicillins, cephalosporins and macrolides
J01(CR + DC + DD+(F-FA01)): broad-spectrum antibacterials
J01(DD + DE): third- and fourth-generation cephalosporins
J01CR: combination of penicillins, including beta-lactamase inhibitor
J01-J07X: antiinfectives for systemic use
J01MA: fluoroquinolones
MoH: Ministry of Health
MRSA: methicillin-resistant *Staphylococcus aureus*
MSSA: methicillin-sensitive *Staphylococcus aureus*
NDM: New Delhi metallo-β-lactamase
NHF: National Health Fund
NICU: Neonatal Intensive Care Units
NRCAST: National Reference Centre for Antimicrobial Susceptibility Testing
SE: Southern European
URTI: upper respiratory tract infections
WHO: World Health Organization
